# Current status and trend of laparoscopic right hemicolectomy for colon cancer

**DOI:** 10.1002/ags3.12373

**Published:** 2020-07-18

**Authors:** Takeru Matsuda, Kimihiro Yamashita, Hiroshi Hasegawa, Masako Utsumi, Yoshihiro Kakeji

**Affiliations:** ^1^ Division of Gastrointestinal Surgery Department of Surgery Kobe University Graduate School of Medicine Kobe Japan; ^2^ Division of Minimally Invasive Surgery Department of Surgery Kobe University Graduate School of Medicine Kobe Japan

**Keywords:** colon cancer, laparoscopic right hemicolectomy, open surgery, robotic surgery

## Abstract

Laparoscopic right hemicolectomy (LRH) is utilized worldwide as one of the standard surgical treatments for right‐sided colon cancer. However, there have been issues concerning its applicability, techniques, and trend. The present study aimed to elucidate the current status and trend of LRH by reviewing literature focusing on important issues associated with this surgery. Based on previous studies, LRH most likely provides better short‐term outcomes and similar oncological outcomes compared to open surgery. Despite the increasing use of robotic approach in this surgery, it seems to have always been associated with longer operative times and greater hospital cost with limited advantage. Intracorporeal anastomosis seems to improve short‐term outcomes, such as quicker recovery of bowel function, compared to extracorporeal anastomosis. However, it does not contribute to shorter hospital stay. With regard to dissection technique, various approaches, and landmarks have been advocated to overcome the technical difficulty in LRH. This difficulty is likely to be caused by anatomical variation, especially in venous structures. The superiority of one approach or landmark over another is still argued about due to the lack of large‐scale prospective studies. However, deep understanding both of anatomical variation and characteristics of each approach would be of extreme importance to minimize adverse effects and maximize patient benefit after LRH.

## Graphical abstract

The present study aimed to elucidate the current status and trend of laparoscopic right hemicolectomy by reviewing literature focusing on important issues associated with this surgery.

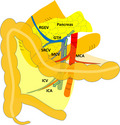

## 1 INTRODUCTION

Since the first report of laparoscopic surgery for colorectal cancer in 1991,^1^ its popularity has been increasing worldwide based on evidence that laparoscopic surgery provides better short‐term outcomes and similar oncological outcomes compared with open surgery, supported by previous large randomized clinical trials (RCTs).^2^, ^3^, ^4^, ^5^ Laparoscopic right hemicolectomy (LRH) is one of the most common procedures in colorectal cancer surgery, which is employed for right‐sided colon cancer. However, it has several unique issues unlike other colorectal cancer surgeries. There were several different approaches for lymph node dissection (i.e. medial‐to‐lateral, lateral‐to‐medial, and cranial‐to‐caudal) and reconstruction (i.e. intracorporeal and extracorporeal). Furthermore, there are anatomical variations in vascular structures encountered during LRH, and various landmarks have been proposed for a successful surgery. The optimal extent of lymphadenectomy is also being discussed. In fact, open surgery is still preferred and performed in a considerable number of cases despite the strong evidence demonstrating the superiority of laparoscopic surgery in terms of short‐term outcomes.^6^ In recent years, utilization of robotic approach has been reported as an alternative to LRH.

Based on the annual report of the Japanese National Clinical Database (NCD), right hemicolectomy has been more widely and frequently performed by non‐board‐certified surgeons in gastroenterology compared with low anterior resection.^7^ However, the 30‐day mortality rate is consistently higher in right hemicolectomy than low anterior resection. The rate of laparoscopic surgery in right hemicolectomy is also markedly lower than that in low anterior resection according to the Japanese NCD.^8^ These findings might reveal some clinical problems to be analyzed or discussed on LRH.

Thus, its applicability, techniques, and trend are chaotic, although LRH is popular and widespread as one of the standard surgical treatments for right‐sided colon cancer. The present article aimed to elucidate the current status and trend of LRH by reviewing literature focusing on important issues associated with this surgery.

## 2 LAPAROSCOPIC VS OPEN APPROACH

Previous major randomized trials have already reported the superiority of short‐term outcomes and non‐inferiority of oncological outcomes of laparoscopic surgery for colon cancer compared with open surgery.^2^, ^3^, ^4^, ^5^ Even for advanced colon cancer with clinical stage II/III, better short‐term outcomes and similar overall survival in laparoscopic surgery compared with open surgery with a low conversion rate of 5.4% were demonstrated by the Japan Clinical Oncology Group (JCOG) 0404 trial.^9^, ^10^ When focusing on LRH, similar outcomes in favor of laparoscopic surgery were demonstrated by recent studies.^11^, ^12^, ^13^, ^14^, ^15^, ^16^ In 2015, Arezzo et al reported that the mortality and morbidity rate was lower in the laparoscopic group than the open group (1.2% vs 3.4%; *P* = .031; and 16.8% vs 24.2%; *P* = .007, respectively) based on their systematic review on 3049 patients.^11^ Moreover, Rausa et al reported in their meta‐analysis a significantly higher risk of overall complication and reoperation in the open surgery group compared to the total laparoscopic surgery group.^14^ Hospital stay was also markedly longer in the open group than in the total laparoscopic group. On the other hand, the contrasting results were demonstrated by Jurowich et al using a propensity score analysis of patient data in the DGAV StuDoQ|ColonCancer registry in Germany.^6^ In their study, the laparoscopic surgery resulted in significantly shorter hospital stay (odds ratio [OR], 0.55; 95% Confidence Interval [CI], 0.47‐0.64) and significantly longer operative time (OR, 2.32; CI, 1.98‐2.71) than the open group. There was no significant difference with regard to the rate of postoperative complications, such as anastomotic insufficiency, ileus, and reoperation, between the groups. However, in the original database prior to propensity score matching, only 18.7% of patients (935 of 4062 patients) received laparoscopic surgery, and the conversion rate to open surgery was 16.5%, which was considerably high. Therefore, LRH offers better short‐term outcomes and comparable oncological outcomes compared to open surgery, as long as its surgical quality is assured.

## 3 LAPAROSCOPIC VS ROBOTIC APPROACH

In contrast, the usefulness of laparoscopic vs robotic right hemicolectomy is still uncertain, although a growing number of comparative studies on that topic have been published in recent years.^17^, ^18^, ^19^, ^20^, ^21^, ^22^, ^23^ The first comparative study of the outcome of laparoscopic vs robotic right hemicolectomy was reported by Rawlings et al in 2007.^24^ In their retrospective study, the total operative time was significantly longer in the robotic surgery group (n = 17) than the laparoscopic surgery group (n = 15) (218.9 min vs 169.2 min; *P* = .002). The robotic surgery group demonstrated a significant increase in total operating room (OR) cost, OR personnel cost, OR supply cost, and OR time cost. The first RCT of robotic vs laparoscopic right colectomy was reported by Park et al in 2012.^25^ In their study, hospital stay, operative complications, postoperative pain score, resection margin clearance, and number of harvested lymph nodes were similar in both groups. The operative time was longer, and the overall hospital cost was higher in the robotic group (195 min vs 130 min; *P* < .001; and USD, $12,235 vs $10,320; *P* = .013, respectively). They concluded that robotic right colectomy provided no benefit to justify greater cost. Recent review by Solaini et al demonstrated similar outcomes, such as longer operative time and higher hospital cost in the robotic surgery.^21^ The most recent meta‐analysis compared open surgery, laparoscopic‐assisted surgery, total laparoscopic surgery (with intracorporeal anastomosis), and robotic right hemicolectomy to assess the short‐term outcomes.^14^ Based on their analysis, the overall complication rate was similar between robotic and total laparoscopic surgery but higher in open and laparoscopic‐assisted surgery than robotic surgery. The operative time was similar between robotic and total laparoscopic surgery, and hospital stay was significantly longer in laparoscopic‐assisted surgery than robotic surgery. Their meta‐analysis revealed that the short‐term outcomes following robotic and total LRH were superior to standard laparoscopic and open surgeries. Nevertheless, the use of robotic approach for right hemicolectomy is still argued about due to its association with longer operative times and greater hospital cost with limited advantage of its use. Therefore, laparoscopy is currently the most commonly utilized approach in right hemicolectomy.

## 4 DISSECTION APPROACH

**FIGURE 1:**
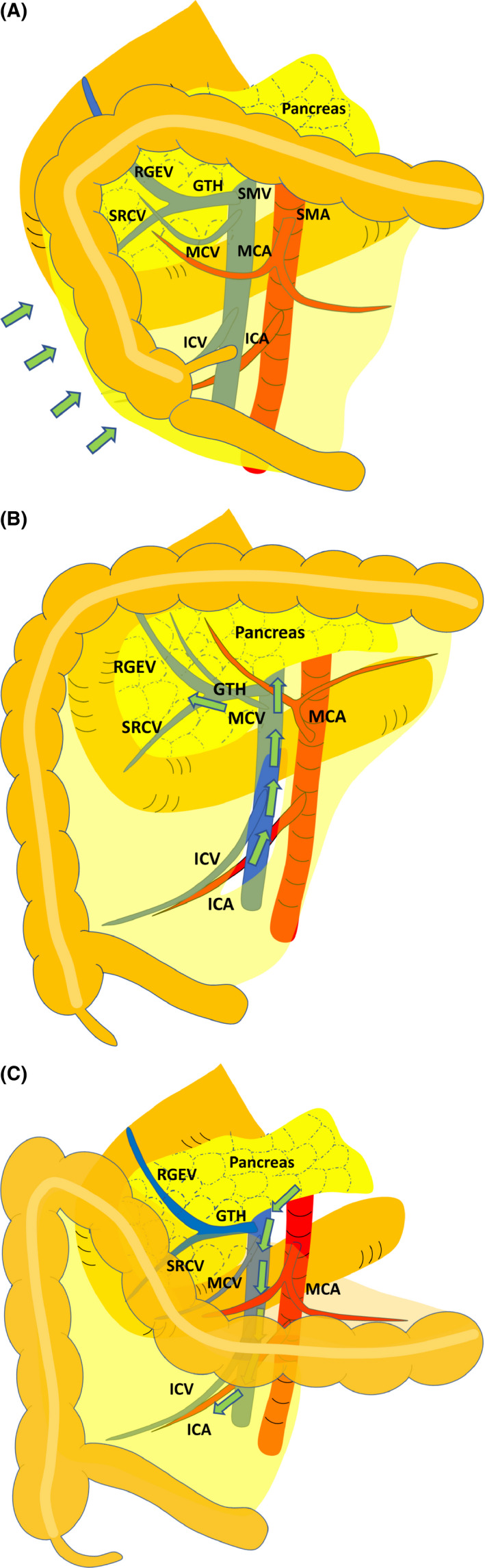
Approaches in laparoscopic right hemicolectomy. A, A lateral‐to‐medial approach. B, A medial‐to‐lateral approach. C, A cranial‐to‐caudal approach. GTH, gastrocolic trunk of Henle; ICA, ileocolic artery; ICV, ileocolic vein; MCA, middle colic artery; MCV, middle colic vein; RGEV, right gastroepiploic vein; SMA, superior mesenteric artery; SMV, superior mesenteric vein; SRCV, superior right colic vein

Laparoscopic dissection in right hemicolectomy was originally initiated by using a lateral‐to‐medial approach to reproduce the same steps usually performed in conventional open surgery, where the white line of Toldt's fascia is incised first and the vascular pedicles divided last (Figure 1A).^26^ Thereafter, some experts in laparoscopic colorectal surgery introduced a medial‐to‐lateral approach, which involves the division of the vascular pedicle first, followed by mobilization of the mesentery, and finally division of the colon from the white line of Toldt's fascia (Figure 1B).^27^, ^28^, ^29^, ^30^ In 2004, a consensus of the European Association for Endoscopic Surgery stated that medial‐to‐lateral approach was recommended as the preferred approach for laparoscopic colectomy.^31^ According to the retrospective comparative study by Rotholtz et al in 2009, the operative time for LRH was significantly shorter in the medial‐to‐lateral approach than in the lateral‐to‐medial approach (148.6 min vs 185.6 min; *P* = .009); however, the morbidity and mortality did not differ between the groups.^30^ A meta‐analysis comparing these two approaches in laparoscopic colorectal surgery demonstrated similar results, including the advantages of shorter operative time and possibly lower conversion rate in the medial approach.^32^, ^33^ However, in LRH, a cranial‐to‐caudal approach, another representative approach, has been recently developed and utilized, which first involves opening of the omental bursa, early exposure and dissection of medial colic vessels, and subsequent lymph node dissection along the surgical trunk in either top‐to‐bottom or bottom‐to‐top manner (Figure 1C).^34^, ^35^ Although the comparative study on the medial, lateral, and cranial approaches is very limited, Li et al performed a network meta‐analysis comparing these three different approaches in LRH in 2017.^36^ According to their data, the lateral approach needed shorter postoperative flatus recovery time than both medial and cranial approaches. The length of hospital stay was also shorter in the lateral approach compared with the medial approach. The cranial approach achieved less postoperative complications, including anastomotic leak, ileus, wound infection, pneumonia, acute urinary retention, wound hernia, and postoperative hemorrhage than the medial approach. Interestingly, the operative time did not differ between the groups. Generally, the lateral‐to‐medial dissection in laparoscopic colectomy is technically demanding due to limited operative space and insufficient maneuverability of the straight laparoscopic forceps. To overcome these difficulties in LRH, new laparoscopic approaches, such as pincer approach, artery‐first approach, and uncinate process first approach are emerging.^37^, ^38^, ^39^, ^40^ Suprapubic bottom‐to‐up approach has also been developed for robotic right hemicolectomy.^41^ Taken together, the superiority of one approach over another is still debated, and prospective studies on a large scale would be needed for further evaluation.

## 5 ANASTOMOTIC TECHNIQUE

It is also important to ascertain whether anastomotic technique after LRH, intracorporeal anastomosis (IA), or extracorporeal anastomosis (EA) is superior. Previous retrospective studies reported controversial results. Some investigators have demonstrated earlier recovery of bowel function, lower morbidity, and shorter hospital stay in patients with IA compared to those with EA,^42^, ^43^, ^44^ while other investigators did not illustrate any significant advantages in IA.^45^, ^46^, ^47^ Recently, two important RCTs comparing these two techniques after LRH were conducted.^48^, ^49^ One double‐blinded RCT in Italy by Allix et al demonstrated a quicker recovery of bowel function after IA than EA (gas, 2 days; interquartile range [IQR], 2‐3 vs 3 days; IQR, 2‐3, *P* = .003; stool, 4 days; IQR, 3‐5 vs 4.5 days; IQR, 3‐5, *P* = .032].^48^ However, the median length of hospital stay was similar between the groups (6 days; IQR, 5‐7 vs 6 days; IQR, 5‐8; *P* = .839). The median operative time was also comparable between the groups. No significant differences were observed in the length of skin incision, morbidity, reoperation rate, and readmission rate between the two groups. Another RCT from Spain reported the superiority of IA to EA in various short‐term outcomes.^49^ Although surgery with IA had a longer operative time, it resulted in shorter wound length (6.7 cm vs 8.7 cm; *P* < .001), quicker recovery of digestive function (2.3 days vs 3.3 days; *P* = .003), lower incidence of paralytic ileus (13% vs 30%; *P* = .022), less postoperative analgesia, less gastrointestinal bleeding (3% vs 14%; *P* = .031), and less grade I and II complications according to Clavien‐Dindo classification. There was no difference in terms of the duration of hospital stay between the groups (5.7 days for IA vs 6.6 days for EA; *P* = .194). Just recently, Widmar et al reported that the 1‐year estimated incisional hernia rate was 12% for extracorporeal and 2% for intracorporeal anastomoses (*P* = .007) after robotic right colectomy.^50^ As a result, IA seems to improve short‐term outcomes, such as earlier recovery of bowel function, although it does not contribute to shorter hospital stay.

## 6 D3 OR CME/CVL?

Since complete mesocolic excision with central vascular ligation (CME/CVL) was proposed for colon cancer surgery, CME/CVL has been regarded as the principal procedure for laparoscopic colon cancer surgery.^51^, ^52^, ^53^, ^54^ In this method, the tumor is resected using the embryologic tissue planes along with the entire regional mesocolon in an intact peritoneal and fascial lined package.^53^ In LRH, using this method, lymph nodes along the ileocolic, middle colic, and superior mesenteric vessels and peripancreatic and gastroepiploic lymph nodes should be dissected.^53^, ^54^ Previous studies have demonstrated the improved oncological or pathological results after this surgery compared to conventional surgery.^54^, ^55^, ^56^ A population‐based cohort study employing 1069 patients with right‐sided colon cancer in Denmark reported that the 5.2‐year cumulative incidence of recurrence was 9.7% in the CME group compared with 17.9% in the control group, and that the absolute risk reduction of CME after 5.2 years was 8.2% (95% CI, 4.0‐12.4; *P* = .00015).^56^ On the other hand, in Japan, D3 dissection is recommended for advanced colon cancer with cT3/4 or cN + according to the Japanese Society for Cancer of the Colon and Rectum (JSCCR) guidelines.^57^ The JSCCR defines lymph node classification as follow: pericolic lymph nodes –—lymph nodes along the marginal arteries and near the bowel wall; intermediate lymph nodes—lymph nodes along the ileocolic, right colic, and middle colic arteries; and main lymph nodes—lymph nodes at the origin of the ileocolic, right colic, and middle colic arteries.^58^ In Japanese D3 dissection, pericolic, intermediate, and main lymph nodes are removed along with CME. Kanemitsu et al demonstrated the optimal Japanese D3 dissection in right hemicolectomy and reported that 5‐year disease‐specific survival of patients with stages I, II, and III cancer were 100.0%, 94.5%, and 85.0%, respectively.^59^ In their study, the 5‐year overall survival (OS) and disease‐specific survival (DSS) of patients with metastases to N3 nodes were 36.4% for both, and 5‐year OS and DSS of patients with metastases to N2 nodes were 77.6% and 83.5%, respectively. The oncological validity of Japanese D3 for colon cancer has also been demonstrated by several studies in the past, similar to CME/CVL.^9^, ^60^, ^61^, ^62^ According to the interesting study employing 4034 patients with stage III colon cancer (right = 1618, left = 2416) by Kataoka et al, the right‐sided cancers more frequently invaded main lymph nodes than left‐sided lesions (8.5% vs 3.7%; *P* < .001) and the proportion of patients with a skipped pattern of lymphatic spread was higher in right than in left colon cancer (13.7% vs 9.0%; *P* < .001).^63^ These results suggest D3 would be required for clinical stage II/III right‐sided colon cancer.

An excellent comparative study of Japanese D3 and CME/CVL was conducted by West et al,^64^ concluding that Japanese D3 specimens were significantly shorter (162 mm vs 324 mm; *P* < .001), resulting in a smaller amount of mesentery (8,309 mm^2^ vs 17,957 mm^2^; *P* < .001), compared with the European CME/CVL. The distance from the high vascular tie to the bowel wall was comparable. Although the number of dissected lymph nodes for right‐sided tumor was smaller in Japanese D3 than European CME/CVL (median, 24 vs 32; *P* = .004), the number of positive nodes did not differ significantly (median, 0 vs 1; *P* = .410). Kobayashi et al focused on the comparison of surgical specimen for stage III colon cancer between Japanese D3 and European CME/CVL and reported similar results.^65^ Although the rigid distinction of these two concepts seems difficult, both are based on the same oncological principles.

Besides the oncological aspects, feasibility and safety are also important issues. CME/CVL or D3 is considered technically more difficult than non‐CME/CVL or D2. According to the Copenhagen Complete Mesocolic Excision Study (COMES) by Danish Colorectal Cancer Group, intraoperative injury to other organs was more common in CME operations (9.1% vs 3.6% for non‐CME resection; *P* < .001), including more superior mesenteric vein injuries (1.7% vs 0.2%; *P* < .001).^66^ On the other hand, the multicenter RCT in Russia revealed that the 30‐day postoperative morbidity rate was 47% in the D2 group and 48% in the D3 group, with a risk ratio of 1.04 (95% CI, 0.68 to 1.58, *P* = .867).^67^ Postoperative recovery, complication and readmission rates did not differ between the groups. Thus, the feasibility and safety of CME/CVL or D3 is still controversial.

As proposed by Sammour et al, optimal D2 lymphadenectomy (removal of pericolic and intermediate nodes to the right side of the SMV) with standard high ligation as a minimum standard and selective CVL (D3 lymphadenectomy) in selected patients, such as cN + disease, would be an ideal stance for colon cancer at the moment.^68^

## 7 LANDMARK

One of the main reasons for technical difficulties of LRH is anatomical complexity, including the wide variation in vascular anatomy and embryological adhesion of the transverse mesocolon to the adjacent organs. It also contributes to immature standardization of LRH procedure. To overcome these issues, numerous studies on reliable landmarks for successful LRH have been published.

Among such literature, the gastrocolic trunk of Henle (GTH) seems to be the most reliable landmark, since the use of GTH was advocated for LRH by Bergamaschi et al.^69^ Yamaguchi et al reported a 69% presence rate of GTH in 58 cadavers and that GTH was formed with the right colic vein in 27.5% of cases and with the middle colic vein in 75% of cases.^70^ He et al reported the characteristics of GTH based on intraoperative findings during LRH from 371 patients.^71^ In their study, GTH was present in 97.8% of patients (363 of 371), and it was most commonly formed with the right colic vein alone. They emphasized the relatively short length of GTH (8.5 mm on average, ranging from 2 to 30 mm), which might carry a risk of bleeding. A recent review by Peltrini et al revealed that GTH was found in 74% of cadaver studies and in 86% of radiological studies.^72^ The superior right colic vein (SRCV) joins the right gastroepiploic vein and the anterior superior pancreaticoduodenal vein, forming GTH in most cases.

On the other hand, Sun et al proposed the use of the ileocolic vein as an anatomical landmark during LRC due to its presence in 100% of patients while GTH was present in approximately 80%.^73^ Ignjatovic et al recommended SRCV as a landmark instead of GTH because GTH is not easily accessible due to its tight relations with the right colon arteries.^74^ Komolafe et al use the head of the pancreas as a landmark for mobilization of the transverse colon with proximal isolation and ligation of the middle colic artery.^75^ Garcia‐Granero et al provided the fusion fascia of Fredet as an essential embryological landmark during LRH, which corresponds to the plane between the ascending mesocolon and the visceral duodenal‐pancreatic peritoneum.^76^

Although all landmarks seem useful and reliable, the anatomical knowledge of vascular and organ structures would be the most important for a secured surgical procedure despite the considerable anatomical variability, as advocated by Peltrini et al.^72^

## 8 CONCLUSION

Although LRH is one of the most common surgeries for colorectal cancer, it comes with various issues currently debated among colorectal surgeons. These issues seem to be mainly caused by complex anatomy encountered during LRH, including a wide variation in vascular anatomy. Substantial excellent surgical techniques have been developed to overcome these problems, and more novel approaches or devices will be invented in the future. However, deep understanding of both anatomical variation and characteristics of each approach would be essential to minimize adverse effects and maximize patient benefits after LRH.

## DISCLOSURE

Conflict of Interest: The authors have no conflicts of interest.
